# Barriers and facilitators on the HIV care continuum among adults living with HIV in high-income countries: a scoping review protocol

**DOI:** 10.1186/s13643-022-02097-x

**Published:** 2022-10-15

**Authors:** Gwang Suk Kim, Youngin Kim, Min Kyung Park, Sang A Lee, Youngjin Lee, Mi-So Shim

**Affiliations:** 1 Mo-Im Kim Nursing Research Institute, College of Nursing, Yonsei University, 50-1 Yonsei-ro Seodaemun-gu, Seoul, 03722 Republic of Korea; 2 Yonsei Evidence Based Nursing Centre of Korea: A JBI Affiliated Group, 50-1 Yonsei-ro Seodaemun-gu, Seoul, 03722 Republic of Korea; 3 Department of Nursing, Graduate School of Yonsei University, Seoul, Republic of Korea; 4 Robert and Donna Manning College of Nursing and Health Sciences, University of Massachusetts Boston, 100 William T. Morrissey Blvd., Boston, MA 02125-3393 USA; 5 College of Nursing and Brain Korea 21 FOUR Project, Yonsei University, 50-1 Yonsei-ro Seodaemun-gu, Seoul, 03722 Republic of Korea; 6 College of Nursing, Keimyung University, 1095 Dalgubeol-daero, Dalseo-gu, Daegu, 42601 Republic of Korea

**Keywords:** HIV, Care continuum, Barriers and facilitators, Scoping review

## Abstract

**Background:**

As the life expectancy of people living with HIV increases with the advancements in antiretroviral treatment, the continuity of long-term therapy and health care for people living with HIV has gained more importance. However, the estimated proportion of people living with HIV who have access to treatment or are virally suppressed is unsatisfactory. Therefore, it is necessary to build strategies to improve treatment continuity by identifying the barriers and facilitators that affect the HIV care continuum. To enable this, we will conduct a scoping review to explore the barriers and facilitators related to the care continuum in high-income countries for adults living with HIV.

**Methods:**

The review question will be identified based on the JBI guidelines for the development of scoping review protocols. Studies exploring the barriers to and facilitators of the HIV care continuum among adults living with HIV in high-income countries will be included in this review. A literature search will be conducted on the databases (platform) of MEDLINE (Ovid), Cumulative Index to Nursing and Allied Health Literature (EBSCO), Embase (Ovid), and the Cochrane Central Register of Controlled Trials (Cochrane Library). Four researchers will screen articles for inclusion and subsequently build a charting form and collate the data to provide results.

**Discussion:**

The results of this scoping review will provide comprehensive evidence for the barriers and facilitators to be considered in the care continuum of people living with HIV. Importantly, the results will provide insight for healthcare providers and researchers to develop interventions and research the continuity in caring for people living with HIV.

**Supplementary Information:**

The online version contains supplementary material available at 10.1186/s13643-022-02097-x.

## Background

The number of people living with HIV (PLHIV) has increased globally, estimated at 38 million as of 2019 [1]. This increase, attributable to the number of people accessing treatment, is due to the improved effectiveness of antiretroviral therapy (ART) [2]. In addition, with the development of therapeutic drugs, such as a decrease in the size or number of drugs and their side effects, the medication adherence and quality of life of PLHIV have improved [3]. According to the results of a study analyzing the life expectancy of 24,768 patients with HIV and 257,600 uninfected population in the USA, the life expectancy gaps between PLHIV and uninfected people are gradually decreasing [4]. Patients diagnosed with HIV at the age of 20 have been reported to live until their early 70s if they receive ART early and receive continuous treatment [4, 5]. Thus, it is necessary to ensure the continuity of long-term treatment and health care for patients with HIV, similar to patients with other chronic diseases.

Several phases of HIV treatment have been studied: (1) from the first diagnosis to the treatment initiation [6, 7], (2) to a few months of ART to verify its effectiveness for PLHIV [6, 8], (3) to treatment retention, and (4) a long-term shift to improving health behaviors for those with stable HIV status [9, 10]. In 2015, as a global agreement on these phases, the Joint United Nations Programme on HIV/AIDS (UNAIDS) proclaimed the target of achieving “90-90-90” by 2020: 90% of PLHIV diagnosed, 90% of those diagnosed antiretrovirally treated, and 90% of those treated virally suppressed [11]. This led to the consensus that, as a chronic disease, it was more important to maintain the HIV care continuum—from the first diagnosis to long-term health care—to emphasize the specific periods in HIV care.

Despite the 90-90-90 indicator, among all patients with HIV, the estimated proportion of patients who knew their HIV status was 84% [67–98%], patients who accessed treatment was 73% [56–88%], and patients who were virally suppressed was 66% [53–79%] in 2020 [1]. These proportions indicate a current need for interventions to maintain the HIV care continuum. Fox and Rosen suggested stages for the HIV care continuum, including linkage to care, treatment initiation, and early and lifelong retention in care, and emphasized the importance of improving the continuity of care by preventing the loss of patients from each stage of care [12]. Thus, providing appropriate interventions to prevent loss of patients from care at each stage is necessary. According to previous systematic reviews, peer-led interventions, financial incentives, and patient navigation interventions have been attempted as interventions to improve the care continuum of patients with HIV [13–15]. In particular, patient navigation intervention had positive effects on linkage to care and retention in care by removing barriers affecting patient treatment [15, 16]. To develop such interventions, comprehensive identification of the barriers and facilitators that affect each stage of the HIV care continuum is necessary.

Previous reviews reported that the common barriers to HIV care were HIV-related stigma, alcohol and substance abuse, and depressive symptoms; social support and resilience were reported as facilitators of HIV care [17, 18]. Specifically, not accepting a HIV diagnosis and not recognizing the need for treatment due to the absence of specific health problems were barriers to linkage to care [19]. Additionally, healthcare providers’ attitudes and ART inaccessibility were reported as barriers to retention in care [20, 21]. However, it is difficult to find reviews that comprehensively present the barriers and facilitators influencing each care continuum stage. In addition, there are differences in HIV incidence, prevalence, and ART coverage between high- and low-income countries [22]. Divergencies were also reported in the risk factors related to the HIV care continuum between high- and low-income countries [18]. The results of the meta-analysis revealed that being single and younger were significant risk factors for low ART adherence in low-income countries but not in high-income countries [18]. Therefore, national income level should be considered as a context when examining barriers and facilitators on the HIV care continuum.

The HIV care continuum emphasizes maintaining the health of PLHIV and preventing transmission through adherence to treatment [23, 24]. However, previous studies were limited to patients with HIV in specific populations [17, 25] or only included a specified time point of the HIV care continuum [26]. Additionally, in a systematic review, there is a limitation in that neither quantitative nor qualitative studies are comprehensively analyzed and integrated [27]. A scoping review is useful when the knowledge on a topic has not been comprehensively reviewed, or the subjects of exploration have a complex and heterogeneous nature [28]. In addition, it is useful for identifying how research on a specific topic or field is conducted, as well as the existing knowledge gaps, and serves as a precursor to a systematic review [29]. Through this scoping review, we aim to provide comprehensive knowledge about barriers and facilitators that should be considered in the care of adults living with HIV in clinical fields and identify existing knowledge gaps.

## Methods

We developed this protocol based on the JBI guidelines for the development of scoping review protocols [30], the scoping review framework proposed by Arksey and O’Malley, and recommendations by Levac and colleagues [31, 32]. In addition, the scoping review will be reported in accordance with the Preferred Reporting Items for Systematic reviews and Meta-Analyses extension for Scoping Reviews (PRISMA-ScR) checklist [33]. The detailed research process and content are as follows:

### Stage 1: identifying the review question

The review question in this review will help to identify the barriers and facilitators affecting the care continuum of adults living with HIV. This study will explore barriers and facilitators on the care continuum, such as those affecting linkage to care, medication adherence, and retention in care, to provide basic data for interventions to improve care continuum of patients with HIV in community and/or clinical settings. The primary review question is as follows: what are the barriers to and facilitators of the care continuum (e.g., linkage to care, medication adherence, and retention in care) of adults living with HIV that are reported in the existing literature?

### Stage 2: identifying related studies

The research team will develop inclusion criteria using the “population-concept-context” (PCC) framework based on the JBI guidance for the development of scoping review protocols. The inclusion criteria based on the PCC framework is shown in Table 1. Peer-reviewed journal articles on quantitative and qualitative studies that are written in English or Korean will be included in this scoping review.

**Table 1:** Inclusion criteria based on the PCC framework

**Participants**	
Adults over the age of 18 living with HIV	
**Concept**	
Studies about the barriers and facilitators affecting the HIV care continuum, including linkage to care, medication adherence, and retention in care	
**Context**	
Studies conducted in clinical or population-based settings	
Studies conducted in high-income countries	

As a research team, with the assistance of a librarian, we will develop a comprehensive search strategy (Supplemental material 1). We plan to conduct a literature search on the databases of MEDLINE (Ovid), Cumulative Index to Nursing and Allied Health Literature (EBSCO), Embase (Ovid), and the Cochrane Central Register of Controlled Trials (Cochrane Library) that will include peer-reviewed articles published in English or Korean between January 2013 and September 2022. The search period will be set to begin from 2013 because the guidelines to start ART changed from when the number of CD4 immune cells fell below a certain level to immediately after the diagnosis of HIV infection [34, 35]. The “retention in pre-ART care until treatment eligibility” stage has been excluded from the HIV care continuum by the new guidelines [12]. Therefore, we will focus on identifying barriers and facilitators on the new HIV care continuum.

### Stage 3: study selection

**Fig. 1:**
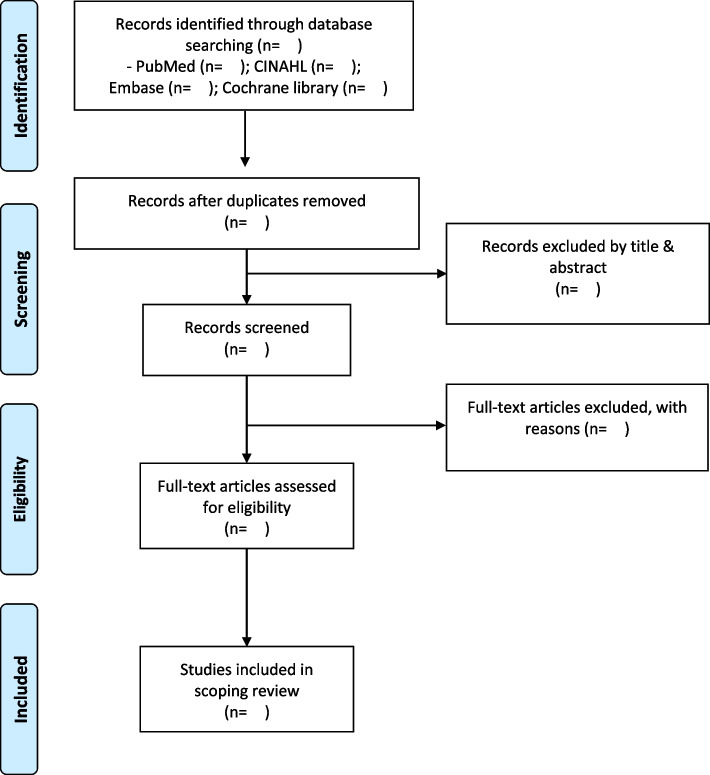
PRISMA flow diagram

First, the results retrieved from each database will exclude duplicate articles using the EndNote X9.2 program. Three researchers will screen titles and abstracts to exclude articles that do not meet eligibility criteria. Second, a review of full-text articles will determine which to include in the final analysis. Last, we will report this process using the PRISMA flow diagram (Fig. 1). To ensure systematic processing, we will conduct adequate discussions and training prior to screening and selection and report the progress through weekly research meetings and discussion of results.

### Stage 4: charting the data

The research team will build a charting form through discussions, including characteristics of the screened articles and outcomes. Table 2 shows the content expected to be included in the charting form. Prior to charting, researchers will be provided sufficient training on the methods of data abstraction from experts with extensive experience in scoping review research. Subsequently, each researcher will perform data abstraction of five randomly selected articles and discuss the results, modifying the charting form if necessary. Through this process, this review will proceed with charting of all articles based on the final confirmed charting form. If the need arises for a collaborative discussion on specific aspects during the charting process, each researcher will record and present these at weekly review meetings. Additionally, if necessary, the research team will request the corresponding authors, via email, for the full articles.

**Table 2:** Data extraction form

**Characteristics of the included studies**	
First author	
Year of publication	
Country	
Study design	
Study population	
**Care continuum outcome and measurement**	
Included care continuum outcomes	
Measurements of the care continuum outcomes	
**Barriers and facilitators**	
Identified barriers to the care continuum	
Identified facilitators to the care continuum	

### Stage 5: collating, summarizing, and reporting the results

The barriers and facilitators will be classified according to the pillars of the care continuum (e.g., linkage to care, medication adherence, and retention in care) presented in each study. Afterward, the number of studies will be analyzed in which barriers and facilitators affecting each pillar of the care continuum have been reported. Through this process, the research team can identify (1) the frequency of barriers and facilitators that have been shown to affect the care continuum pillars and (2) results that require further exploration of the barriers to and facilitators of care continuum pillars (e.g., young age was reported as a barrier to the HIV care continuum in one study; however, in another study, it was reported to be a facilitator of the HIV care continuum). Furthermore, the synthesized results will enable the research team to present the framework on barriers and facilitators in the HIV care continuum and propose implications for future research, practice, and policy.

### Stage 6: consultation exercise

Levac et al. suggested collecting evidence from a stakeholder consultation to ensure the methodological rigor of scoping reviews [32]. To identify evidence from the stakeholder consultation, we will gather expert opinions from the PLHIV, who are consumers of this exploration topic, and nurses and physicians who provide health services across the care continuum. A list of barriers and facilitators configured through the five-step process will be provided to the experts to evaluate the validity of each item of the list based on their own experience and knowledge.

## Discussion

This scoping review aims to identify barriers and facilitators that affect improving the continuity of care for PLHIV. The purpose of this scoping review is to facilitate a comprehensive analysis and integration of existing evidence on the specific details of barriers and facilitators affecting the HIV care continuum. The results confirmed through this review will provide the content to constitute strategies designed to reduce barriers and improve facilitators in the continuity of care by healthcare providers in clinical fields. In addition, these results can also be useful as evidence for policies and guidelines for PLHIV and provide insight into future research topics and directions to reduce the knowledge gap regarding the care continuum of PLHIV.

One of the limitations of our review protocol is that as we will only include articles published in English and Korean in the analysis, selection bias may occur. Additional limitations that may arise in the process of this study will be described in the full-text scoping review.

## Supplementary Information

**Additional file 1.** Search strategy.

## Data Availability

All data generated or analyzed during this study will be included in the published scoping review article.

## Abbreviations

ART: Antiretroviral therapy
HIV: Human immunodeficiency virus
PLHIV: People living with HIV
PCC: Population-Concept-Context
