# Supplementary figures and images for: Barriers and facilitators on the HIV care continuum among adults living with HIV in high-income countries: a scoping review protocol
Source: Syst Rev. 2022 Oct 15;11:222. doi: 10.1186/s13643-022-02097-x (PMC9569070; doi:10.1186/s13643-022-02097-x)

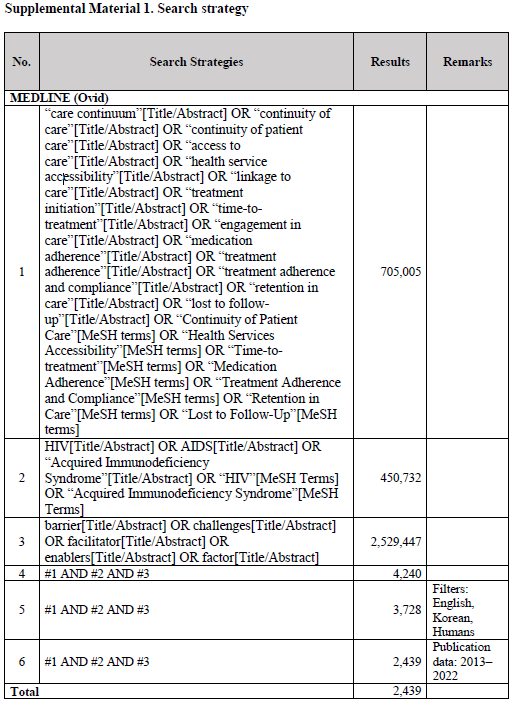

Supplement: Supplementary file 1 — Additional file 1. Search strategy. [file 13643_2022_2097_MOESM1_ESM.png]
